# Access to oral rehydration solution and zinc supplementation for treatment of childhood diarrhoeal diseases in Sudan

**DOI:** 10.1186/s13104-020-05268-y

**Published:** 2020-09-10

**Authors:** Sagad Omer Obeid Mohamed, Mansour Osman Alhaj Alawad, Asaad Ahmed Mohammed Ahmed, Ahmed Abdallah Ali Mahmoud

**Affiliations:** Faculty of Medicine, University of Khartoum, Alqasr Avenue, P.O. Box 102, Khartoum, Sudan

**Keywords:** Diarrhoea, Children, ORS, Zinc, Epidemiology, MICS

## Abstract

**Objectives:**

The decline in diarrhoeal disease-related mortality globally has been attributed to the use of oral rehydration solution (ORS) and zinc supplementation. However, data on ORS and zinc supplementation in Sudan are scarce. We aimed to investigate the access to ORS and zinc treatments and the associated factors, through the analysis of the latest available data from Sudan-Multiple Indicator Cluster Survey (MICS)-2014 obtained from the United Nations Children’s Fund (UNICEF).

**Results:**

A total of 14,081 children were included in this analysis. During the 2 weeks preceding the survey, 29.3% of these children had a diarrhoeal disease. Only 18.9% and 14.8% of these children had received ORS and zinc supplements, respectively. Whereas children from the higher wealth index groups were more likely to receive ORS treatment (fourth group: AOR = 1.301; 95% CI 1.006–1.682), children from rural areas were less likely to receive ORS treatment (AOR = 0.666; 95% CI 0.552–0.803) and zinc supplements (AOR = 0.603; 95% CI 0.500–0.728). The results indicate the existence of unequal access to treatment of childhood diarrhoeal diseases among children under 5 years in Sudan.

## Introduction

Diarrhoeal diseases create a global health burden with significant morbidity and mortality among children under 5 years in low and middle-income countries [1–3]. About half-a-million diarrhoeal disease-related deaths occur annually, with the highest rates of under-5 year child diarrhoea-related mortality recorded in sub-Saharan Africa and South Asia [1, 4, 5]. Despite improvements in standards of living, diarrhoeal diseases still account for significant economic and social losses [6]. Eradication of most of the preventable deaths due to diarrhoeal diseases could be achieved by scaling up cost-effective interventions [7, 8].

The decline in the total diarrhoeal diseases-related mortality globally has been attributed to the increased use of oral rehydration solution (ORS), zinc supplementation, improved nutrition, hygiene, and sanitation [10–13]. The World Health Organization (WHO) and United Nations Children’s Fund (UNICEF) recommended ORS and zinc supplementation as a primary treatment for diarrhoeal diseases in children because ORS can reduce diarrhoeal deaths significantly and zinc supplementation can reduce the duration, severity, and recurrence of diarrhoeal diseases in children in the 2 to 3 months following its use [9–13].

Data on childhood diarrhoeal diseases and ORS and zinc supplementation in Sudan are scarce and most of the studies on diarrhoeal disease in Sudan were hospital or small-community-based studies. This study was conducted to investigate diarrhoeal diseases prevalence, access to ORS and zinc treatments, and their associated factors among under 5 years of age children in Sudan.

## Main text

### Methods

#### Source of data

In this study, we analysed the latest available data (5th round) of the Sudan-Multiple Indicator Cluster Survey (MICS), which was conducted from August to December 2014 at the national level, covering all 18 states of Sudan [14–16]. MICS was conducted by the Central Bureau of Statistics and Ministry of Health as part of the global MICS programme. Several international collaborators, including the WHO, World Food Programme (WFP), and the United Nations Population Fund (UNFPA), funded the Sudan-MICS [14–16]. UNICEF developed the global MICS programme in order to collect internationally comparable data on several indicators of the health condition of women and children. These data allow countries to generate conclusive evidence for use in policies and to monitor progress towards the internationally accepted commitments [14–16].

Participants of the MICS-2014 were recruited using a two-stage stratified clustered sampling method. The sample size for Sudan-MICS 2014 was calculated as 18,000 households, and the number of households occupied was 17,142. Of these, 16,801 households were successfully interviewed. In the interviewed households, 14,751 children under 5 years were listed in the household questionnaires. The questionnaires were completed for 14,081 of them (response rate: 95.5%). The questionnaires were pretested and administered to mothers or caretakers of the children. Further details regarding the MICS are published in reports elsewhere [14–16].

#### Study variables

The variables included in this study were selected based on the availability of the data collected. The study examined the following variables: child age (infants/1–2 years/2–3 years/3–4 years/4–5 years); sex (boy/girl); area of residence (urban/rural); mother education (none/primary/secondary/higher); and household wealth index quintile (poorest/second/middle/fourth/richest). The primary outcome variables: a self-reported history of diarrhoea in the past 2 weeks (Yes/No), receiving ORS treatment (Yes/No), and receiving zinc supplementation (Yes/No).

#### Data analysis

The weighted analysis was conducted in two parts. First, we performed a bivariate regression analysis to examine associations between uptake of ORS, zinc treatment, and the selected associated factors. The selected significant variables (*p* value < 0.2) were further entered into the multiple logistic regression models and the results were reported as adjusted odds ratios (AOR), with a 95% confidence interval. All statistical analyses were conducted using SPSS software version 20 (SPSS Inc., Chicago, IL, USA).

### Results

The number of children under 5 years included in the analysis was 14,081. Most of the participants (23.2%) were in the 3–4-year age group, and 21% of participants were infants. Children from urban and rural areas were 27.4% and 72.6%, respectively. The socio-demographic characteristics of the participants are presented in Table 1. From the analysis, 4088 (29.3%) children reported having a diarrhoeal disease in the past 2 weeks. ORS and zinc supplements had been administered to 758 (18.9%) and 593 (14.8%) participants in this category respectively (Table 1).

**Table 1:** Characteristics under-5 year children participated in the study

Variable	Level	Total no	Percentages
Age group	Infants	2964	21.0
	1–2 year	2672	19.0
	2–3 year	2618	18.6
	3–4 year	3268	23.2
	4–5 year	2559	18.2
Sex	Male	7157	50.8
	Female	6924	49.2
Area of residence	Urban	3862	27.4
	Rural	10,219	72.6
Mother education	None	5994	42.6
	Primary	4936	35.1
	Secondary	2152	15.3
	Higher	982	7.0
Wealth index	Poorest	3188	22.6
	Second	3015	21.4
	Middle	2956	21.0
	Fourth	2684	19.1
	Richest	2238	15.9
Having diarrhea in the past 2 weeks	Yes	4088	29.3
	No	9878	70.7
Received ORS	Yes	3251	81.1
	No	758	18.9
Received zinc	Yes	3416	85.2
	No	593	14.8

From the included children, the overall proportion of having a diarrheal illness in the past 2 weeks among under-5 year children was 29.3% (4088 patients). Treatment with ORS and zinc supplementation was provided to 18.9% (758 patients) and 14.8% (593 patients) of the affected children, respectively (Table 1).

#### Results of logistic regression analysis

The binary regression analysis showed that access to ORT for diarrhoeal diseases was associated with the age group, area of residence, and wealth index quintiles. Multiple logistic regression analysis showed that children living in rural areas were less likely to receive ORS treatment than those in the urban areas (OR = 0.666; 95% CI 0.552–0.803), whereas children in the fourth (OR = 1.301; 95% CI 1.006–1.682) wealth index group were more likely to receive ORS treatment than poorest wealth index group (Table 2). Regarding zinc treatment, multiple logistic regression results showed that access to zinc supplements was associated only with the area of residence. Children in rural areas were less likely to receive zinc supplements than those in the urban areas (OR = 0.603; 95% CI 0.500–0.728) (Table 3).

**Table 2:** Multiple logistic regression results of receiving ORS among under-5 year children

Variable	Level	ORS (%)	AOR	95% CI	p value
Age group	Infants	15.7	Ref	–	–
	1–2 year	20.6	1.379	(1.090–1.746)	0.008
	2–3 year	20.3	1.351	(1.057–1.728)	0.016
	3–4 year	20.5	1.413	(1.097–1.819)	0.007
	4–5 year	16.6	1.068	(0.791–1.442)	0.666
Area of residence	Urban	23.8	Ref	–	–
	Rural	16.8	0.666	(0.552–0.803)	0.000
Wealth index	Poorest	14.9	Ref	–	–
	Second	18.2	1.234	(0.942–1.616)	0.126
	Middle	20.3	1.296	(0.996–1.686)	0.054
	Fourth	20.8	1.301	(1.006–1.682)	0.045
	Richest	20.1	1.108	(0.822–1.493)	0.502

**Table 3:** Multiple logistic regression results of receiving zinc among under-5 year children

Variable	Level	Zinc (%)	AOR	95% CI	p value
Area of residence	Urban	19.6	Ref	–	–
	Rural	12.8	0.603	(0.500–0.728)	0.000
Mother education	None	14.7	Ref	–	–
	Primary	13.6	0.860	(0.700–1.055)	0.147
	Secondary	16.5	1.002	(0.775–1.295)	0.987
	Higher	17.5	1.014	(0.718–1.431)	0.937

### Discussion

Most of the diarrhoeal-related mortality in children are due to dehydration and loss of electrolytes. Successful management of diarrheal diseases with ORS or increased fluid intake can prevent many of these deaths. The WHO and UNICEF started the integrated Global Action Plan for Pneumonia and Diarrhoea (GAPPD) in 2013 [17]. By 2025, this programme seeks to reduce the incidence of diarrhoea by 75% among children under 5 years, as one of the specific objectives. However, this study revealed an increase from 27.8% prevalence recorded in 2000 [18]. This increase is cautionary and should draw the attention of stakeholders in the health sector in Sudan.

The analysis of data from the previous Sudan-MICS in 2000 revealed that age of a child, area of residence, and wealth index were significantly associated with the outcome [18], which is similar to the findings of the current study. Similar to the findings of other studies from Ethiopia and Iraq [19, 20], our findings indicate that children in younger age groups were more likely to have diarrhoeal diseases. The lower prevalence of diarrhoeal diseases in the oldest age group of these children may be due to acquired natural immunity [20].

In this study, the use of ORT was as low as 18.9%. Similarly, ORS for treatment of diarrhoeal diseases in children has been reported to remain below 50% in many low-income countries, despite available evidence suggesting that scaling up the use of ORS is a cost-effective way to highly reduce preventable child deaths [21]. Children living in rural areas and were less likely to receive ORS treatment and zinc supplementation than those in the urban areas, whereas children in the higher wealth index groups were more likely to receive ORS treatment. Unequal access to treatment of the childhood diarrhoeal diseases among under 5 years children has also been reported in several developing countries [22, 23]. Household wealth has also been reported to be a determinant of the treatment-seeking behaviour for childhood diarrhoeal diseases [23].

Some high-burden countries have established successful scale-up programmes to increase coverage and access rates of ORS and zinc supplementation among target population groups [8, 24]. Paediatric diarrhoeal treatment rates in Kenya increased from 0.8 to 15% in 5 years after permission was given to dispense zinc supplements over-the-counter and government procurement was shifted to co-packaged ORS and zinc. These policies created a competitive market for ORS and zinc supplements and enhanced healthcare provider practices in the management of diarrhoeal diseases [24].

## Limitations

Due to limitations in the data obtained for this analysis, we could not include further analyses of other aspects of the treatment of diarrhoeal diseases such as increased home intake and feeding, antibiotic use for treatment of some the common infectious causes of diarrhoea, such as amoebic dysentery and giardiasis; and inpatient management of severe dehydration and shock. Further studies for the assessment of all of these aspects of diarrhoea treatment are highly recommended.

## Data Availability

The dataset used during this study is available from the corresponding author on reasonable request. The underlying dataset that was further analysed in this study is available from https://mics.unicef.org/surveys.

## Abbreviations

MICS: Multiple indicator cluster survey
WHO: World Health Organization
UNICEF: United Nations Children’s Fund
UNFPA: United Nations Population Fund
WFP: World Food Program
GAPPD: Global Action Plan for Pneumonia and Diarrhoea
